# Attempted Removal of a Large Pheochromocytoma via a Robotic Approach

**DOI:** 10.7759/cureus.70053

**Published:** 2024-09-23

**Authors:** Britiel Bethishou, Debora J Fox-McClary, Susan Wcislak

**Affiliations:** 1 Surgery, Arizona College of Osteopathic Medicine, Midwestern University, Glendale, USA; 2 Colon and Rectal Surgery, Abrazo Community Health Network, Glendale, USA; 3 Endocrine Surgery, Surgical Group of Arizona, Phoenix, USA

**Keywords:** da vinci surgical system, hypertensive emergency, laparoscopic adrenalectomy, open adrenalectomy, pheochromocytoma, tumor resection

## Abstract

A pheochromocytoma is a rare tumor of neuroendocrine origin that secretes hormones like epinephrine, norepinephrine, and occasionally dopamine. The most notable symptom of the release of catecholamines is exceedingly high blood pressure. A 45-year-old male presented with a symptomatic pheochromocytoma of large size. He reported experiencing fatigue, orthopnea, dizziness, headache, and chest tightness with deep inspiration for several months. At the time of presentation, he was found to be in hypertensive emergency and was started on alpha-blockade for several days followed by a beta-blockade for a total of 28 days of anti-hypertensive treatment. During his admission, CT imaging showed his tumor to measure at least 14 cm in length, larger than most. After stabilization of the patient, an attempt was made to resect the tumor robotically, but the procedure was converted to an open left adrenalectomy owing to the size of the tumor and the number of tributaries. The patient tolerated the procedure well and, afterward, his blood pressure normalized. He recovered postoperatively with minimal hypotensive episodes and was started on physical therapy. This report reviews the details and management of this case.

## Introduction

Pheochromocytomas are diagnosed yearly in about 0.8 per 100,000 person-years [1]. They arise from chromaffin cells of the adrenal medulla and secrete catecholamines. About 40% of individuals experience symptoms, which can include hypertension, headache, flushing, and sweating [2]. The average size of a tumor is about 4.9 cm [1]. The size of the tumor, along with other factors like the patient’s body mass index (BMI), germline genetic test results of the tumor, the experience of the operating surgeon, and the likelihood of tumor malignancy affect the surgical approach to be taken. The most common approaches are the transabdominal approach (TA) and the posterior retroperitoneoscopic approach (PRA). Both are done either laparoscopically or robotically in order to be minimally invasive; however, sometimes an open surgical resection is required. Tumors larger than 8 cm are a contraindication for the PRA approach because of the lack of space to work with. Consequently, tumors larger than 5.5 cm are typically approached with open resection because studies show that they are more likely to rupture and lead to pheochromocytomatosis otherwise [2].

At present, a quick search of pheochromocytoma case studies reveals that most tumors average around 3-5 cm, with one case study reporting an 11 cm tumor as “gigantic” [3]. It appears that a pheochromocytoma of 14 cm, as in our patient’s case, is exceedingly rare, and thus the surgical approach to its removal warrants sharing. Here we report a case of a 14.5x8.7x9.5 cm large left adrenal mass, diagnosed as a pheochromocytoma with labs and imaging studies, and its attempted robotic resection.

## Case presentation

The patient was a 45-year-old male with a history of asthma and hypertension, who was on amlodipine 10 mg per os daily. He was admitted to the intensive care unit (ICU) after presenting to the emergency room with a hypertensive emergency. He saw his primary care physician earlier in the day complaining of one week of fatigue, orthopnea, tachycardia, headache, dizziness, chest tightness aggravated by deep inspiration, and high blood pressure. At that time, he was noted to have a blood pressure of 208/78 mmHg and was directed to the emergency room where computed tomography (CT) angiography of the chest was obtained to rule out pulmonary embolism. The CT was negative for pulmonary embolism and instead revealed multiple cystic lesions and multiple nodules of the lungs as well as a left adrenal mass. A CT of the abdomen was then obtained, which revealed a 14.5x8.7x9.5 cm large centrally necrotic left adrenal mass. The presence of an adrenal mass along with the hypertensive emergency prompted pheochromocytoma as a differential diagnosis. The patient was transferred to our facility and admitted to the ICU. He arrived on a diltiazem drip.

Initial vitals included a left arm blood pressure of 150/74, a heart rate of 118 beats per minute, and a respiratory rate of 31. The patient had a body mass index (BMI) of 24.95 at that time. On physical exam, his abdomen was soft, non-tender, and mildly distended. He also had 1+ pitting edema of the lower extremities. He appeared well-developed but tired and was tachycardic. Otherwise, he was in no acute distress. Initial labs indicated WBC of 11.4 K/µL, hemoglobin of 10 g/dL, MCV of 78.5 fL, d-dimer pf 2.27 µ/mL, BUN of 26 mg/dL, creatinine of 2.5 mg/dL, sodium of 128 mEq/L, potassium of 3 mEq/L, BNP of 452 pg/mL, and troponin of 0.11 ng/mL. These lab values are shown in Table 1. Twelve days after arrival, the patient's urine normetanephrine and metanephrine results were elevated to four times the upper limit of normal. The patient was also found to be chromogranin A positive.

**Table 1:** Initial lab results of the patient upon presentation to our ICU. ICU, intensive care unit

Test	Result	Unit	Range
Hemoglobin	10	g/dL	10.5-14
MCV	78.5	fL	72-99
WBC	11.4	K/µL	4.5-11
Sodium	128	mEq/L	135-145
Potassium	3	mEq/L	3.5-5.2
BUN	26	mg/dL	6-20
Creatinine	2.5	mg/dL	0.57-1.0
Troponin	0.11	ng/mL	0-0.04
BNP	452	pg/mL	<100
D-dimer	2.27	µ/mL	<0.4

**Figure 1:**
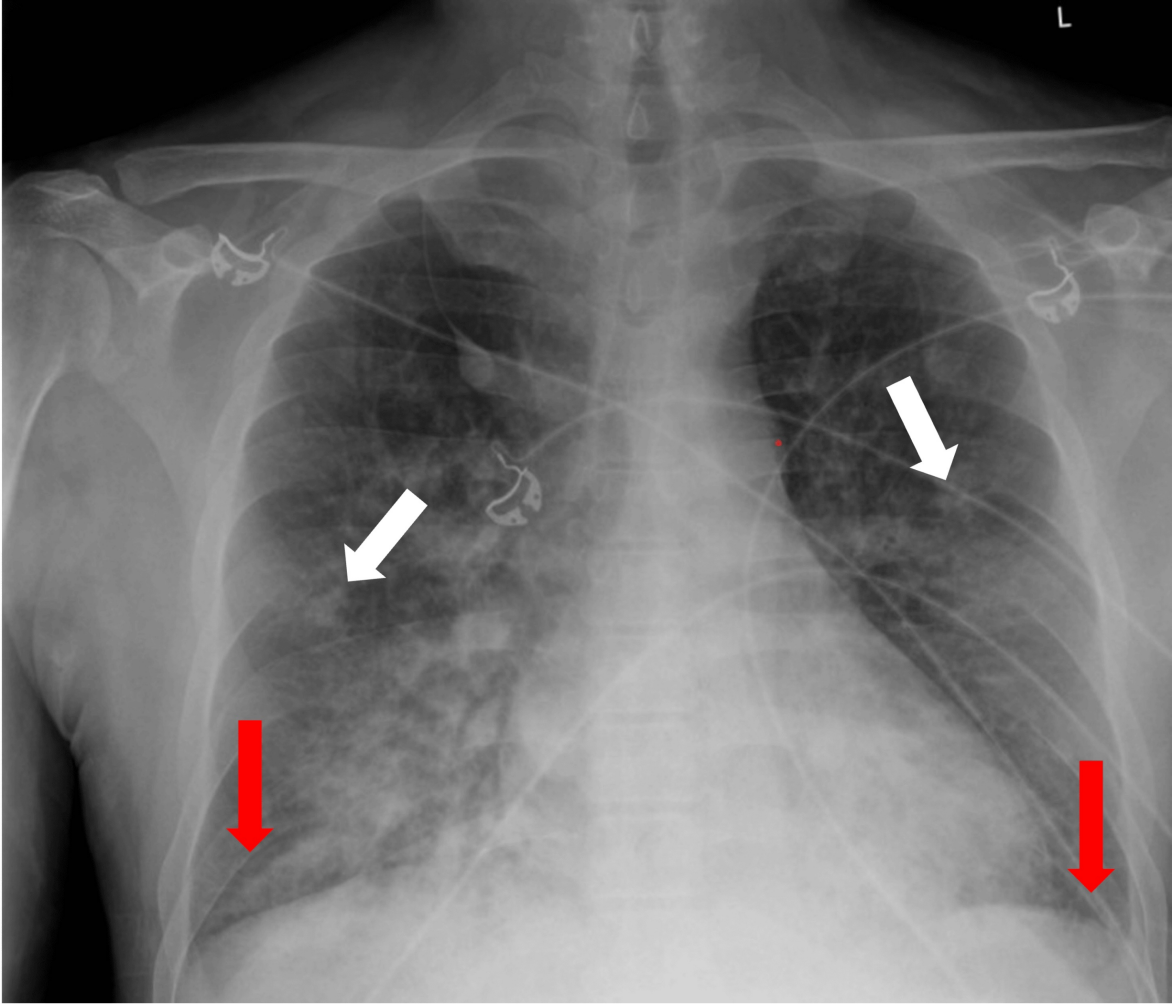
Chest x-ray showing multifocal patchy airspace opacities (white arrows) and bilateral pleural effusions (red arrows), right greater than left, with cystic lung disease.

**Figure 2:**
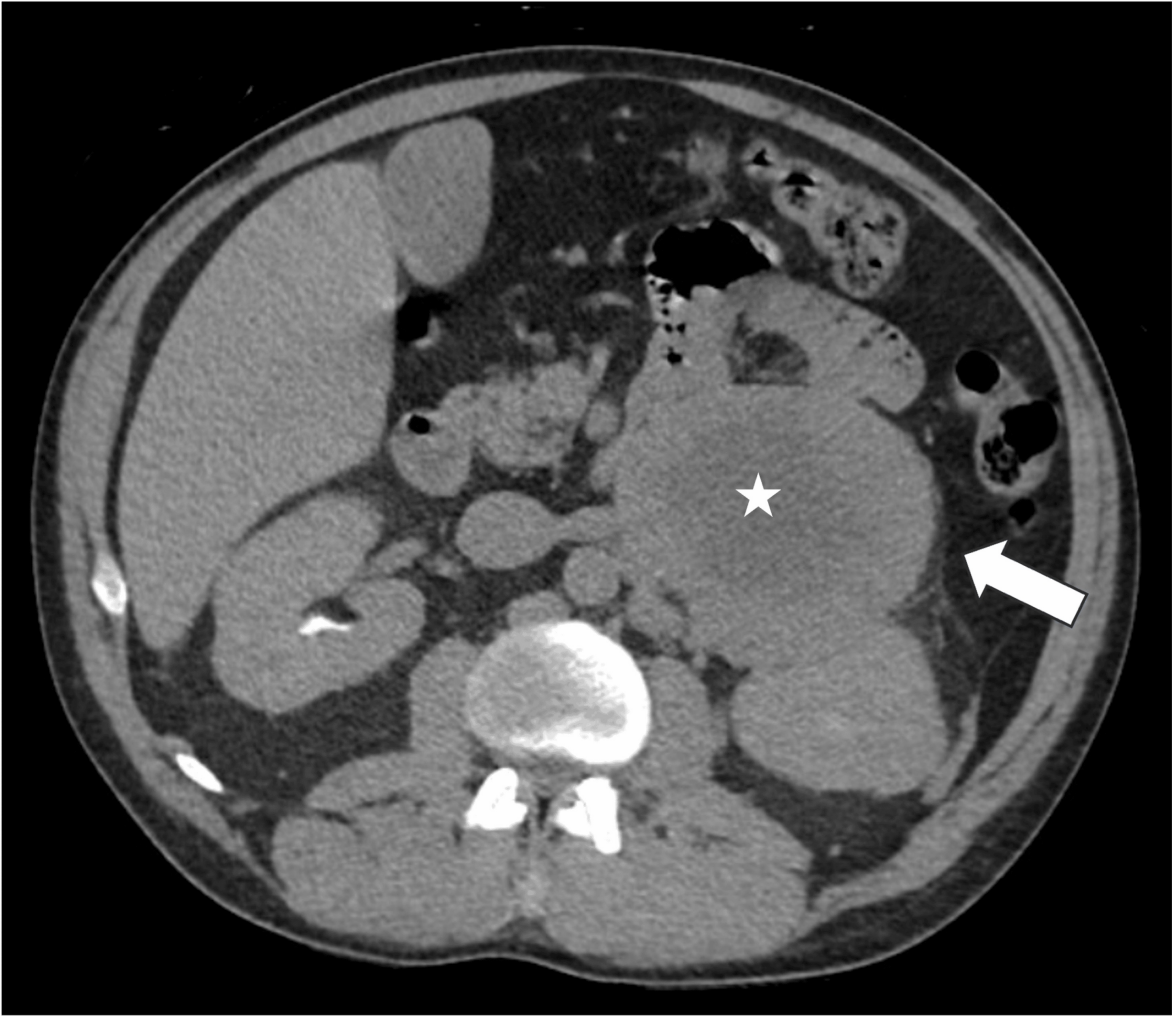
CT abdomen and pelvis identifying the 14.5x8.7x9.5 cm pheochromocytoma (white arrow) with central necrosis (white star). CT, computed tomography

Imaging studies were done, which included a CT abdomen and pelvis without contrast, a CT chest, and a portable chest x-ray. Imaging showed multifocal patchy airspace opacities and bilateral pleural effusions, right more than left, with cystic lung disease, and a large centrally necrotic mass in the left upper quadrant with maximal dimensions of 14.5x8.7x9.5 cm. The left adrenal gland was not definitely identified and the right adrenal gland appeared normal, per radiologist (Figures 1, 2). The patient also had adrenal medullary imaging via iodine-123 meta-iodobenzylguanidine (MIBG) scan, which showed that the large mass in the left upper quadrant showed conspicuous peripheral tracer uptake that is likely to represent pheochromocytoma. 

**Figure 3:**
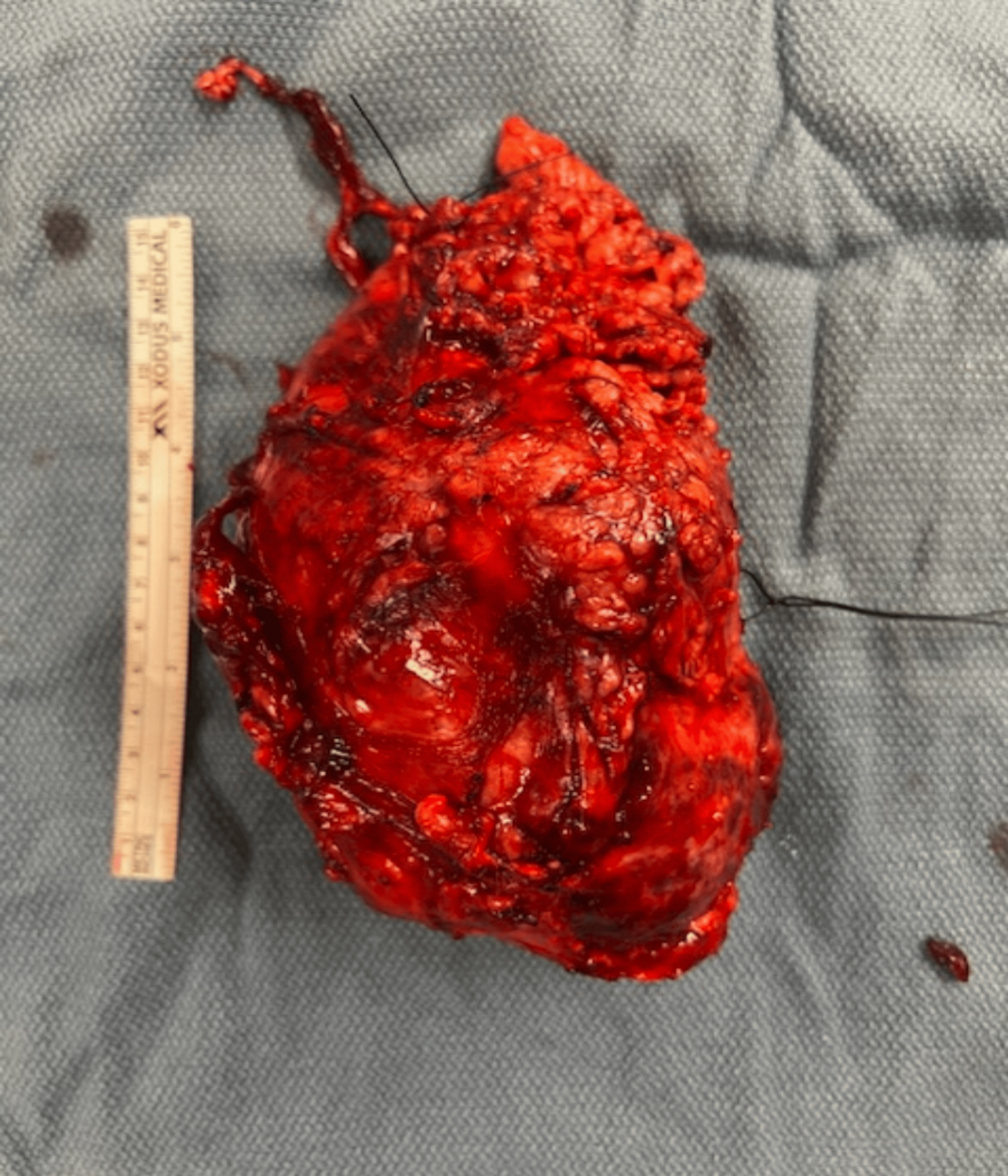
Fully excised, ovoid-shaped tumor with ruler for size.

**Figure 4:**
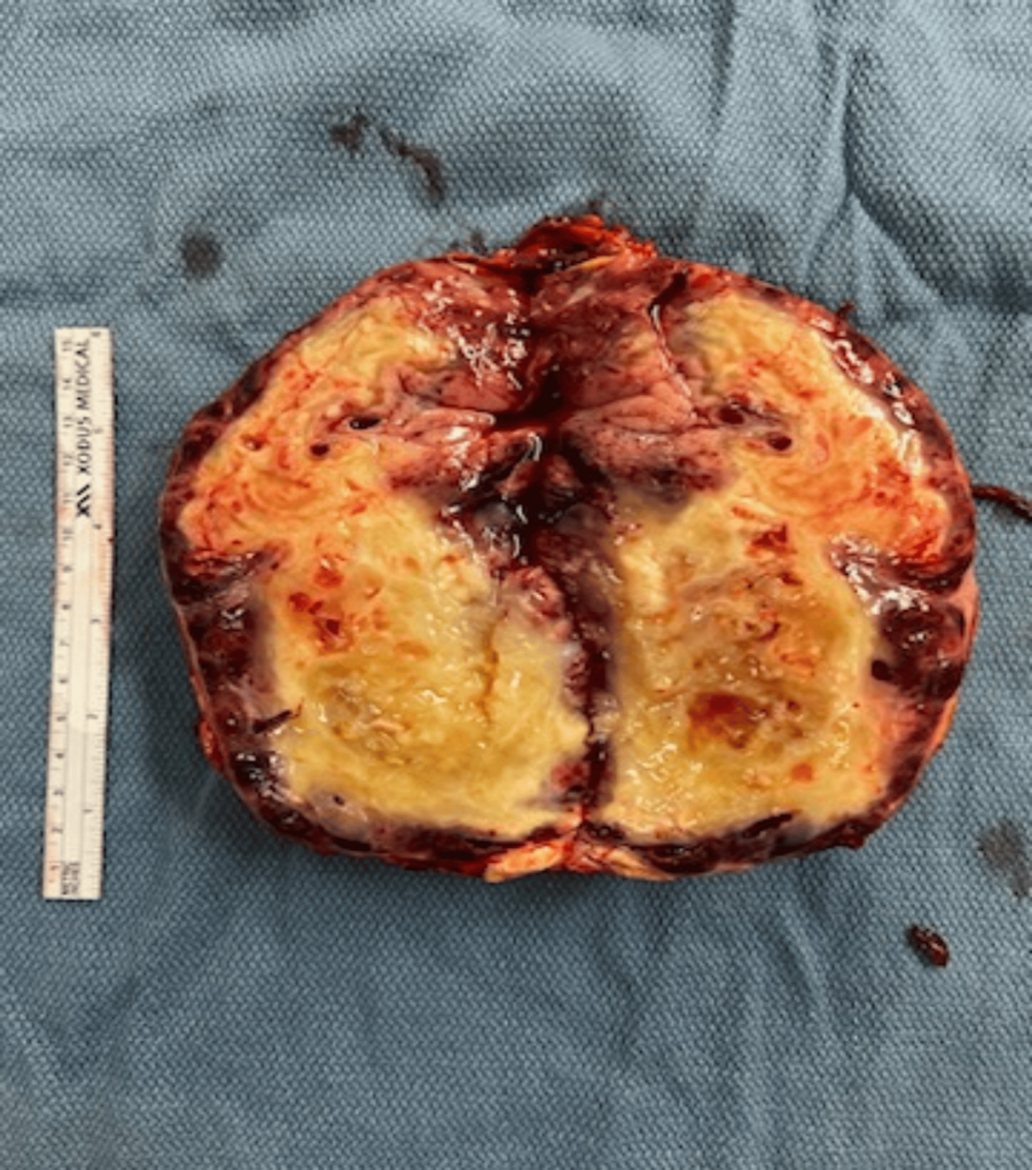
Butterflied specimen showing irregular-shaped, encapsulated mass with central necrosis.

The diltiazem drip was discontinued and the patient was started on nicardipine and doxazosin instead with a goal of systolic blood pressure <120 mmHg, per consulting endocrinologist. Nicardipine was later switched to nitroprusside. A regimen of ceftriaxone and azithromycin was started to cover his respiratory illness. His ICU course included intubation three days after admission for worsening respiratory disease. The patient was extubated two days later and his creatinine slowly improved over the next few weeks. He was begun on carvedilol seven days after starting the doxazosin and was continued on beta-blockade for a little over two weeks until his surgery. When the patient was stable he was finally scheduled for surgery, which took place 28 days after admission to our facility. Surgery was initiated as a DaVinci IX robotic left adrenalectomy but was converted to open left adrenalectomy about two-thirds of the way through the procedure, which lasted for about four hours in total. The attempt to remove the tumor robotically failed because it was too large and had too many tributaries to cauterize/ligate. A robotic approach was taking too much time, resulting in the patient being under anesthesia for longer than necessary. It was simply easier and faster in the end to open the patient up. The pheochromocytoma was removed and examined. Figures 3, 4 below show the mass immediately after removal from the patient’s abdomen. 

The patient tolerated the procedure well and there were no anesthetic complications. The estimated blood loss was 1,700 mL. He was given two units of packed red blood cells and 5 liters of lactated ringers intraoperatively. He was extubated and taken to the post-surgical ICU. The patient showed no evidence of recall. Post-operative vital signs were stable. All anti-hypertensive medications were discontinued. Physical and occupational therapy was continued with the patient after his procedure. One episode of hypotension with systolic in the 70s mmHg was noted during a session of physical therapy the morning following his procedure, but the patient’s blood pressure continued to be in the low-normal range afterward. Orthostatic hypotension was noted on post-operative day one as well. Cardiology, nephrology, and endocrinology continued to follow the patient. His electrolytes were monitored, and he was continued on intravenous fluids. His diet was advanced as tolerated, and his postoperative pain was well controlled. On post-operative day two he was found to be hypertensive again in the 150s-160s mmHg systolic with worsening renal function. He was started on hydrocortisone and his blood pressure improved. He was discharged on post-operative day five with oxycodone for pain management and plans for continued physical therapy, cardiology follow-up, and pulmonary follow-up for his cystic lung disease. By the time of discharge, his blood pressure had normalized.

## Discussion

Pheochromocytomas can develop due to inherited germline mutations or can be sporadic. About 40% of cases are inherited while the other 60% are sporadic [4]. Inherited conditions that predispose to pheochromocytoma include von-Hippel Lindau (VHL) and multiple endocrine neoplasia (MEN) 2A or 2B, to name a few. Our patient’s genetic testing has yet to result at the time of this report. Eight percent of pheochromocytomas of a sporadic nature present with metastasis at the time of diagnosis [4]. The MIBG scan of our patient was negative for metastasis. He had a sporadic tumor, which grew to a large size, and was symptomatic for over a year and did not metastasize. His amlodipine failed to control his blood pressure and his symptoms eventually drove him to seek medical care.

Our patient was found to be anemic, hypokalemic, and hyponatremic. He was also found to have an acute kidney injury, likely owing to dehydration. The elevated troponin and d-dimer were inferred to be due to stress cardiomyopathy from the pheochromocytoma tumor, which simply mimicked acute coronary syndrome, especially since the previously mentioned imaging did not show any pulmonary embolism [5].

Our patient’s tumor was larger than most pheochromocytomas. While smaller tumors, which are less symptomatic, can be managed with alpha and beta blockers, a tumor of this size that induces symptoms in the patient requires surgery. Tumors larger than 5.5 cm typically require an open approach to remove to avoid morbidity [1]. There have been some studies that suggest that the laparoscopic approach, rather than the open approach, leads to less release of catecholamines during surgery [6]. However, as our patient’s pheochromocytoma was very large and had many tributaries to ligate, the benefit of avoiding prolonged time under anesthesia and increased blood loss outweighed the risk of releasing catecholamines via direct manipulation of the tumor.

Prior to surgery, the patient’s blood pressure was optimized and his intravascular volume returned to normal. This is typically done with a seven-day course of alpha-blockade followed up with beta-blockade. At least 10-14 days of antihypertensive treatment should be undertaken in the preoperative period [1]. Our patient received doxazosin for alpha-blockade followed by carvedilol for beta-blockade. His blood pressure was in the 120s-130s mmHg systolic prior to surgery. In the postoperative period, the patient was evaluated for hypotension since their source of excessive catecholamines had just been removed. Some patients require vasopressors and fluids up to 48 hours after tumor removal to control their hypotension [7]. Our patient received fluids when he was hypotensive on post-operative day one but normalized afterward and even had some hypertension a few days later.

In one similar case report, a woman was found to have a pheochromocytoma measuring 16 cm in length, larger than our patient’s tumor. Her tumor presented with a significant mass effect on the patient’s kidney; however, the patient herself presented with no hemodynamic instability. Still, like our patient, she was started on doxazosin, and an open adrenalectomy was performed with no intraoperative complications and much less total estimated blood loss of 200 mL [8]. In that case, opting for an open approach from the beginning likely resulted in less blood loss and less total time under anesthesia than if a laparoscopic adrenalectomy was attempted.

## Conclusions

A pheochromocytoma of 14 cm is an unusually large size for that type of tumor. Most become symptomatic and are brought to the attention of medical professionals before reaching that size. For any symptomatic pheochromocytoma, surgery is usually required and usually curative. Though minimally invasive laparoscopic and robotic approaches are preferred more and more these days for most pheochromocytomas, literature shows that an open approach may be more preferential with larger pheochromocytomas to decrease the risk of tumor rupture and catecholamine release. 
